# Supplementary material for: Distance interventions for enhancing preparedness in informal caregivers of older adults: A systematic review protocol
Source: PLoS One. 2024 Sep 26;19(9):e0309162. doi: 10.1371/journal.pone.0309162 (PMC11426524; doi:10.1371/journal.pone.0309162)
Supplement: S1 Appendix — (DOCX) [file pone.0309162.s001.docx]

**Inclusion and Exclusion Form**

| **Category** | **Inclusion criteria** |
| --- | --- |
| **1. Population** | Were there results reported (i.e., intervention effects)^a^ for a sample or subsample of adults informal/family caregivers^b^ (18 years of age or older) of older adults (60 years of age or older)^c^?  **No Yes**  **If the answer is ‘yes’, rate criterion 2.**  **If ‘no’, exclude the study.** |
| **2. Intervention** | Does the study present an entirely distance intervention^d^?  **No Yes**  **If the answer is ‘yes’, rate criterion 3.**  **If ‘no’, exclude the study.** |
| **3. Design** | Is the study design experimental (i.e., randomized experiments) or quasi experimental (i.e., lacks random assignment)^e^?  **No Yes**  **If the answer is ‘yes’, rate criterion 4.**  **If ‘no’, exclude the study.** |
| **4. Comparator** | Does the study have any comparator (any control group such as placebo, usual care, active control, single pre-post)^f^?  **No Yes**  **If the answer is ‘yes’, rate criterion 5**.  **If ‘no’, exclude the study.** |
| **5. Outcome** | Are some of the intervention outcomes aimed at increasing caregiver ‘preparedness’ (or related concepts or surrogate terms of preparedness) to provide care^g^?  **No Yes**  **If the answer is ‘yes’, rate criterion 6**.  **If ‘no’, exclude the study.** |
| **6. Outcome Measure** | Did the authors measure caregiver preparedness (or related concept) as an outcome, using a scale (e.g., preparedness for caregiving scale, capacity scale, confidence scale) or with a single item and report quantitative findings of intervention effect^h^?  **No Yes**  **If the answer is ‘yes’, rate criterion 7.**  **If ‘no’, exclude the study and answer the following (6A).**  6A) Did the study report on a review (scoping, systematic, narrative) of intervention studies that may meet all study inclusion criteria?  **No Yes**  **If answer 6A is Yes, add the study tag “reviews” in Covidence before answering “no”.** |
| **7. Language** | Is the study written in English or Portuguese?  **No Yes**  **If the answer is ‘yes’, include. If ‘no’, exclude the study.** |

**List of Definitions and Operationalizations**

^a^Reported results should include intervention effects observed in a sample or subsample of informal caregivers of older adults. Intervention effects refer to the differences in outcomes observed after implementing the intervention condition (Reichardt, 2019).

^b^Informal caregiving refers to caregiving provided by family members, friends, or other unpaid individuals (Li & Song, 2021). Formal care refers to paid care services provided by a trained professional (Li & Song, 2021). If the study explicitly refers to the included caregiving population as formal in any section, and there is no inclusion of an informal caregiving sample (in a different arm, for example), then the study should be excluded. Studies can include formal caregivers only if they have separate arms for formal and informal caregivers or reported separate results for each. In studies where the included caregiving population (e.g., informal or formal) is not specified in any section of the study, analyze the provided characteristics of the population to judge if the study should be included or excluded.

^c^Only studies that include interventions designed for caregivers of *older adults* will be included. The age of 60 years was selected according to how the United Nations currently refers to ‘older’ people (United Nations, 2019). Therefore, care recipients must be at least 60 years of age. Studies that include care recipients who are 59 years of age or younger, even if they conduct a subgroup analysis by age, should be excluded. Studies can report the ages of care recipients and caregivers in any section of their studies, such as methods or results sections. If no specific age is provided, please examine whether the introduction offers contextual information regarding age. For instance, if the introduction primarily focuses on older adults, this indicates that the study's population consists of older adults as care recipients. Alternatively, if neither age nor any contextual description is provided in the introduction to establish the ages of care recipients, reviewers can still consider including the study if it pertains to dementia, as this condition is commonly associated with aging (World Health Organization, 2022).

^d^Distance intervention refers to interventions delivered without in-person contact between participants and the intervention facilitator(s) (Ibeggazene et al., 2021; Jenkins et al., 2009). An intervention facilitator is the person who delivers the intervention (e.g., the individual who teaches caregivers how to provide care). Outcomes and installation of platforms can still occur in person, provided that the intervention, including explanation/instructions about the platform and provision of printed materials, is delivered entirely remotely. Distance interventions encompass various methods, including, for example, printed materials, telephone calls, mobile applications (apps), text messaging (SMS), social media platforms, websites, chat rooms, video discussions, email exchange, etc. A distance intervention can be delivered as a control condition, as long as the group receiving the distance intervention does not have in-person contact with the intervention facilitator(s). Studies can also have distance intervention in multiple arms, but in this case, it is essential that the study report an effect estimate that pertains to the distance arm (e.g., comparison pre-post, or to another condition).

^e^Studies eligible for inclusion are scholarly articles (published and preprints), theses, or dissertations that aim to estimate treatment effects using experimental or quasi-experimental study designs (Reichardt, 2019). In experimental designs, randomization is crucial, as it involves randomly assigning study participants to treatment conditions. This random allocation can be achieved through methods such as a coin flip, dice roll, computer-generated numbers, or equivalent randomization techniques. In contrast, quasi-experimental designs lack random assignment. Included studies must provide complete research reports; abstracts and trial registrations will be excluded. Pilot studies are eligible for inclusion.

^f^Studies can encompass different types of comparison groups (Meinert, 2012), including:

- Placebo: where participants in the control group receive a placebo treatment, which is an inactive substance or procedure resembling the active treatment but lacking therapeutic effect.
- Usual Care: where participants in the control group receive standard or usual care, representing typical treatment practices.
- Active Control: where participants in the control group receive an alternative treatment established as effective or standard care, enabling comparison with the experimental treatment.
- Single Pre-Post: where the same participants serve as their own control, eliminating the need for a separate control group. Measurements are taken before and after an intervention, but without a separate control group.

^g^Surrogate terms and related concepts of preparedness include: Preparedness (prepared, prepares, preparation), Readiness, Confidence (self-confidence), Competence, Capacity, and Self-efficacy (Dal Pizzol et al., 2023).

- *Preparedness* is defined as the caregiver's self-confidence about their current competence related to the knowledge, skills, and abilities to perform daily tasks, and to handle emotions over time.
- *Readiness* refers to being available and ready to act at any time when the older person returns from hospital to home, including putting one's own needs aside to stand up for the older person whenever required.
- *Confidence* is defined as the caregiver’s own determination of how well they can execute a plan of action in prospective situations, reflecting their understanding about their strengths and weaknesses.
- *Competence* is the adequacy of how caregivers rate their performance as caregivers.
- *Capacity* encompasses caregiver’s knowledge, skills and competencies.
- *Self-efficacy* refers to caregiver's judgments of their capabilities to organize and execute courses of action required to perform caregiving tasks effectively.

If any of the intervention outcomes is a measure of *preparedness (prepared, prepares, preparation), readiness, confidence (self-confidence), competence, capacity, or self-efficacy* to provide care, the study should be included. Oftentimes the concept may be included within the name of the tool (e.g., General Self-Efficacy Scale). However, this is not required for inclusion. Consider the author’s conceptual and operational definition/description of the outcome to determine if the study outcome measures these concepts.

^h^Analyze the scale assigned to measure the outcome described in the last category of this form (outcome). If it is a quantitative scale or a single item that reports quantitative findings of the intervention effect, then the study should be included.

**References**

Dal Pizzol, F. L. F., O’Rourke, H. M., Olson, J., Baumbusch, J., & Hunter, K. (2023). The meaning of preparedness for informal caregivers of older adults: A concept analysis. *Journal of Advanced Nursing*, *00*, 1–17. https://doi.org/10.1111/jan.15999

Ibeggazene, S., Turner, R., Rosario, D., & Bourke, L. (2021). Remote interventions to improve exercise behaviour in sedentary people living with and beyond cancer: A systematic review and meta-analysis. *BMC Cancer*, *21*(1). https://doi.org/10.1186/s12885-021-07989-0

Jenkins, A., Christensen, H., Walker, J. G., & Dear, K. (2009). The effectiveness of distance interventions for increasing physical activity: A review. *American Journal of Health Promotion*, *24*(2), 102–117.

Li, J., & Song, Y. (2021). Formal and informal care. In D. Gu & M. E. Dupre (Eds.), *Encyclopedia of Gerontology and Population Aging* (Vol. 8, pp. 1905–1911). Springer International Publishing. https://doi.org/10.1007/978-3-030-22009-9_847

Meinert, C. L. (2012). *Clinical trials handbook: Design and conduct*. John Wiley & Sons. https://doi.org/10.1002/9781118422878

Reichardt, C. S. (2019). *Quasi-experimentation: A guide to design and analysis*. The Guilford Press.

United Nations. (2019). *World Population Ageing 2019*. https://www.un-ilibrary.org/content/books/9789210045544

World Health Organization. (2022, October 4). *Ageing and health*. https://www.who.int/news-room/fact-sheets/detail/ageing-and-health
