# Supplementary material for: Distance interventions for enhancing preparedness in informal caregivers of older adults: A systematic review protocol
Source: PLoS One. 2024 Sep 26;19(9):e0309162. doi: 10.1371/journal.pone.0309162 (PMC11426524; doi:10.1371/journal.pone.0309162)
Supplement: S3 Appendix — (DOCX) [file pone.0309162.s003.docx]

**Appendix S3**

Search Protocol

**CINAHL**

| Row | Searches | Results |
| --- | --- | --- |
| 1 | ((measur* or evaluat* or scale* or questionnaire* or assess* or instrument*) N3 (prepar* or readiness or ready or confidence or self-confidence or competen* or capacity or abilit* or capabilit* or self-efficacy or skill* or willingness or mutuality or "mastery of care" or knowledge or understand*)) | 128,986 |
| 2 | (((informal or family or familial or spous* or parent*) N4 (care* or caring)) or carer?) | 84,775 |
| 3 | S1 AND S2 | 2,926 |
| 4 | (distance or remote or online or virtual* or digital* or web* or internet* or Telehealth or "tele health" or telenursing or "tele nursing" or "tele medicine" or telemedicine or ehealth or e-health or "mobile app*" or "smart phone*" or "cell* phone" or mhealth or m-health or computer* or "social media" or videoconferenc* or "video conferenc*" or telephone or zoom) | 712,509 |
| 5 | S3 AND S4 | 538 |
| 6 | (child* or infant* or p?ediatric* or adolescen* or teen*) | 1,419,799 |
| 7 | S5 NOT S6 | 331 |

*Note.* No limits were set for this search.


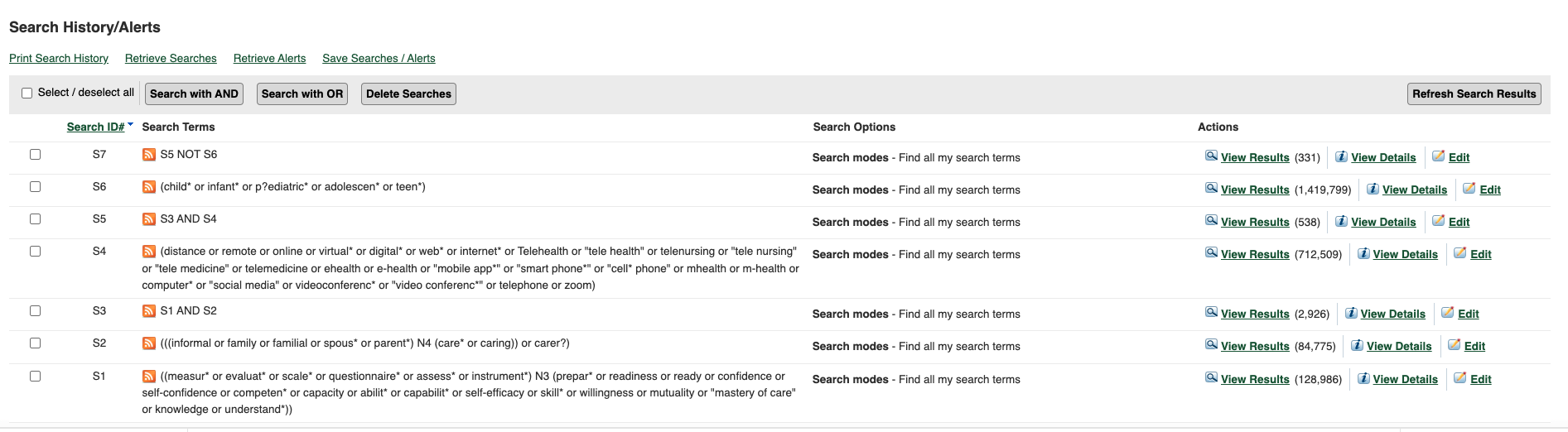


**Cochrane Library**

| Row | Searches | Results |
| --- | --- | --- |
| 1 | ((measur* or evaluat* or scale* or questionnaire* or assess* or instrument*) NEAR/3 (prepar* or readiness or ready or confidence or self-confidence or competen* or capacity or abilit* or capabilit* or self-efficacy or skill* or willingness or mutuality or "mastery of care" or knowledge or understand*)):ti,ab | 34,850 |
| 2 | (((informal or family or familial or spous* or parent*) NEAR/4 (care* or caring)) or carer?):ti,ab | 12,327 |
| 3 | #1 AND #2 | 1,097 |
| 4 | (distance or remote or online or virtual* or digital* or web* or internet* or Telehealth or "tele health" or telenursing or "tele nursing" or "tele medicine" or telemedicine or ehealth or e-health or (mobile NEXT app*) or (smart NEXT phone*) or (cell* NEXT phone) or mhealth or m-health or computer* or "social media" or videoconferenc* or (video NEXT conferenc*) or telephone or zoom):ti,ab | 162,687 |
| 5 | #3 AND #4 | 500 |
| 6 | (child* or infant* or p?ediatric* or adolescen* or teen*):ti,ab | 225,018 |
| 7 | #5 NOT #6 | 300 |

*Note.* No limits were set for this search. Title (ti,) and abstract (ab).

**
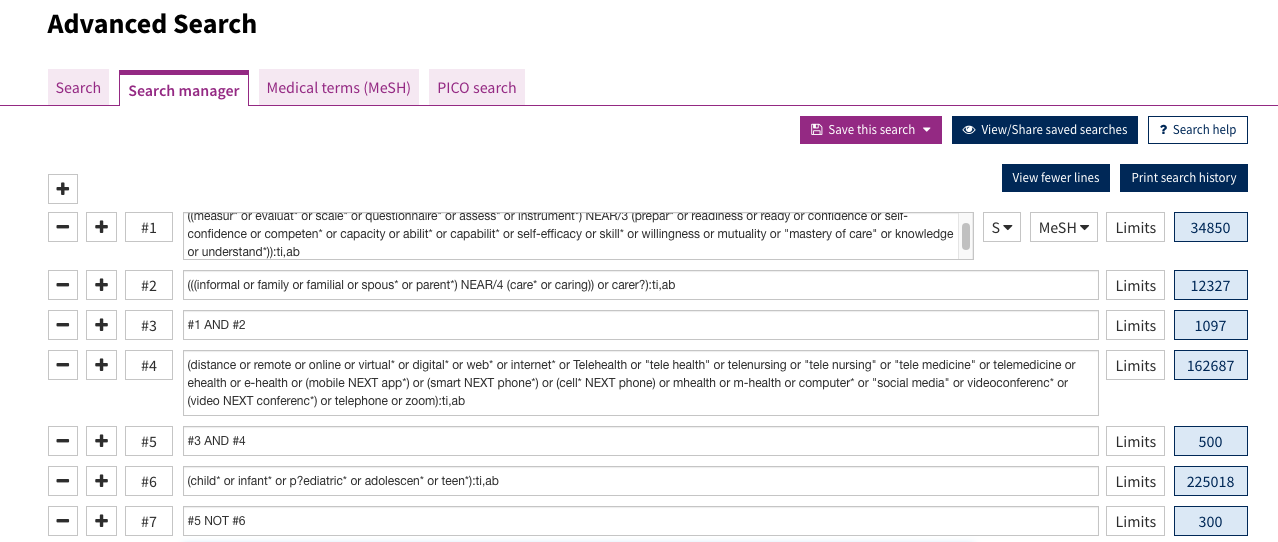
**

**EMBASE**

| Row | Searches | Results |
| --- | --- | --- |
| 1 | ((measur* or evaluat* or scale* or questionnaire* or assess* or instrument*) adj3 (prepar* or readiness or ready or confidence or self-confidence or competen* or capacity or abilit* or capabilit* or self-efficacy or skill* or willingness or mutuality or "mastery of care" or knowledge or understand*)).mp. | 345,148 |
| 2 | (((informal or family or familial or spous* or parent*) adj4 (care* or caring)) or carer?).mp. | 112,372 |
| 3 | 1 AND 2 | 3,477 |
| 4 | (distance or remote or online or virtual* or digital* or web* or internet* or Telehealth or "tele health" or telenursing or "tele nursing" or "tele medicine" or telemedicine or ehealth or e-health or "mobile app*" or "smart phone*" or "cell* phone" or mhealth or m-health or computer* or "social media" or videoconferenc* or "video conferenc*" or telephone or zoom).mp. | 3,540,795 |
| 5 | 3 AND 4 | 706 |
| 6 | (child* or infant* or p?ediatric* or adolescen* or teen*).ti,ab. | 2,990,156 |
| 7 | 5 NOT 6 | 446 |

*Note.* No limits were set for this search. Title (ti,), abstract (ab.), and keywords (.mp).

**
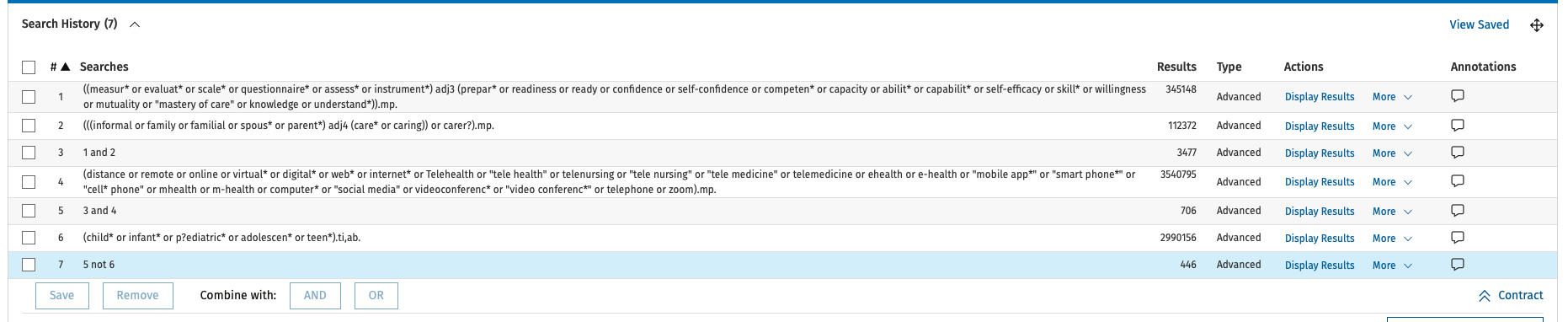
**

**MEDLINE**

| Row | Searches | Results |
| --- | --- | --- |
| 1 | ((measur* or evaluat* or scale* or questionnaire* or assess* or instrument*) adj3 (prepar* or readiness or ready or confidence or self-confidence or competen* or capacity or abilit* or capabilit* or self-efficacy or skill* or willingness or mutuality or "mastery of care" or knowledge or understand*)).mp. | 260,271 |
| 2 | (((informal or family or familial or spous* or parent*) adj4 (care* or caring)) or carer?).mp. | 82,935 |
| 3 | 1 AND 2 | 2,228 |
| 4 | (distance or remote or online or virtual* or digital* or web* or internet* or Telehealth or "tele health" or telenursing or "tele nursing" or "tele medicine" or telemedicine or ehealth or e-health or "mobile app*" or "smart phone*" or "cell* phone" or mhealth or m-health or computer* or "social media" or videoconferenc* or "video conferenc*" or telephone or zoom).mp. | 2,187,212 |
| 5 | 3 AND 4 | 425 |
| 6 | (child* or infant* or p?ediatric* or adolescen* or teen*).ti,ab. | 2,364,238 |
| 7 | 5 NOT 6 | 263 |

*Note.* No limits were set for this search. Title (ti,), abstract (ab.), and keywords (.mp).

**
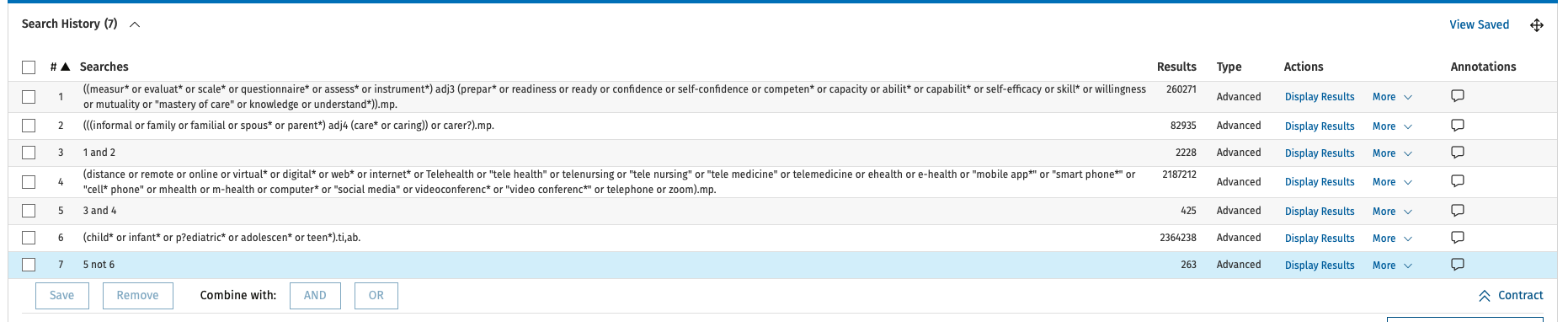
**

**PsycINFO**

| Row | Searches | Results |
| --- | --- | --- |
| 1 | ((measur* or evaluat* or scale* or questionnaire* or assess* or instrument*) adj3 (prepar* or readiness or ready or confidence or self-confidence or competen* or capacity or abilit* or capabilit* or self-efficacy or skill* or willingness or mutuality or "mastery of care" or knowledge or understand*)).mp. | 136,950 |
| 2 | (((informal or family or familial or spous* or parent*) adj4 (care* or caring)) or carer?).mp. | 59,490 |
| 3 | 1 AND 2 | 2,245 |
| 4 | (distance or remote or online or virtual* or digital* or web* or internet* or Telehealth or "tele health" or telenursing or "tele nursing" or "tele medicine" or telemedicine or ehealth or e-health or "mobile app*" or "smart phone*" or "cell* phone" or mhealth or m-health or computer* or "social media" or videoconferenc* or "video conferenc*" or telephone or zoom).mp. | 535,268 |
| 5 | 3 AND 4 | 287 |
| 6 | (child* or infant* or p?ediatric* or adolescen* or teen*).ti,ab. | 1,009,085 |
| 7 | 5 NOT 6 | 197 |

*Note.* No limits were set for this search. Title (ti,), abstract (ab.), and keywords (.mp).

**
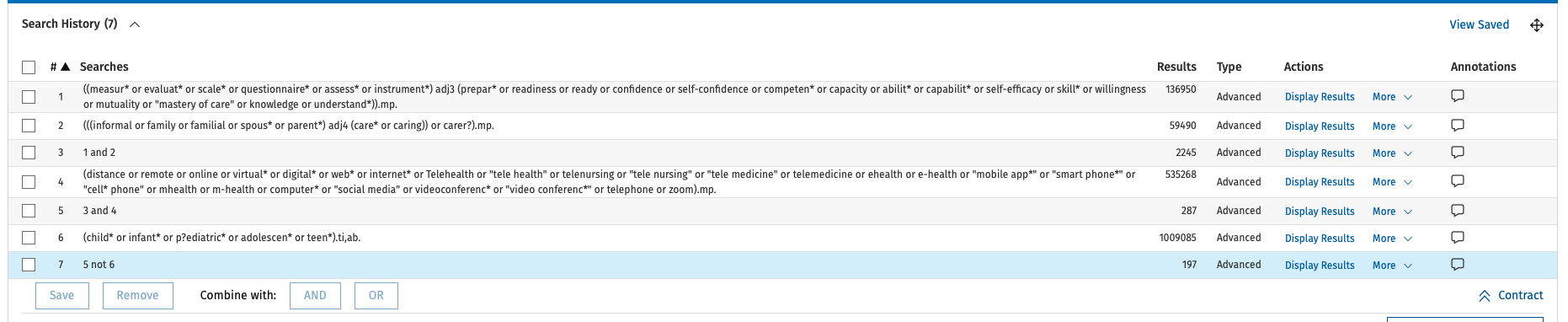
**

**Scopus**

| Row | Searches | Results |
| --- | --- | --- |
| 1 | ( ( TITLE-ABS-KEY ( ( ( measur* OR evaluat* OR scale* OR questionnaire* OR assess* OR instrument* ) W/3 ( prepar* OR readiness OR ready OR confidence OR self-confidence OR competen* OR capacity OR abilit* OR capabilit* OR self-efficacy OR skill* OR willingness OR mutuality OR "mastery of care" OR knowledge OR understand* ) ) ) ) AND ( TITLE-ABS-KEY ( ( ( informal OR family OR familial OR spous* OR parent* ) W/4 ( care* OR caring ) ) OR carer? ) ) AND ( TITLE-ABS-KEY ( distance OR remote OR online OR virtual* OR digital* OR web* OR internet* OR telehealth OR "tele health" OR telenursing OR "tele nursing" OR ehealth OR e-health OR "mobile app*" OR "smart phone*" OR "cell*phone" OR mhealth OR m-health OR computer* OR "social media" OR videoconferenc* OR "video conferenc*" OR telephone OR zoom ) ) ) AND NOT ( TITLE-ABS-KEY ( child* OR infant* OR p?ediatric* OR adolescen* OR teen* ) ) AND ( LIMIT-TO ( DOCTYPE , "ar" ) OR LIMIT-TO ( DOCTYPE , "re" ) ) | 388 |

*
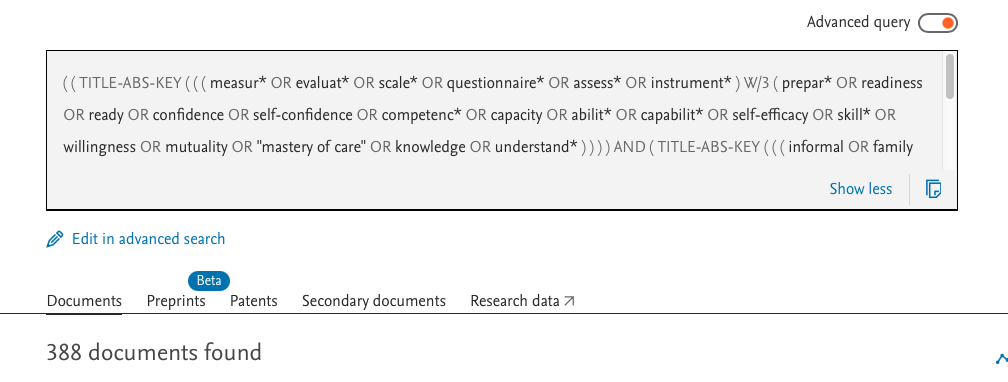
*

**ProQuest Dissertations & Theses Global**

| Row | Searches | Results |
| --- | --- | --- |
| 1 | noft(((measur* or evaluat* or scale* or questionnaire* or assess* or instrument*) NEAR/3 (prepar* or readiness or ready or confidence or self-confidence or competen* or capacity or abilit* or capabilit* or self-efficacy or skill* or willingness or mutuality or "mastery of care" or knowledge or understand*))) AND noft((((informal or family or familial or spous* or parent*) NEAR/4 (care* or caring)) or carer?)) AND noft((distance or remote or online or virtual* or digital* or web* or internet* or Telehealth or "tele health" or telenursing or "tele nursing" or "tele medicine" or telemedicine or ehealth or e-health or "mobile app*" or "smart phone*" or "cell* phone" or mhealth or m-health or computer* or "social media" or videoconferenc* or "video conferenc*" or telephone or zoom)) NOT noft((child* or infant* or p?ediatric* or adolescen* or teen*)) | 65 |

*Note.* No limits were set for this search.

*
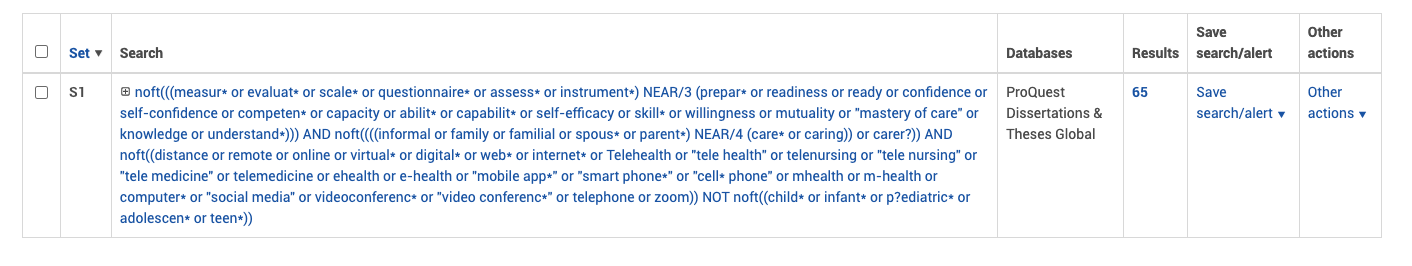
*
