# Supplementary material for: Distance interventions for enhancing preparedness in informal caregivers of older adults: A systematic review protocol
Source: PLoS One. 2024 Sep 26;19(9):e0309162. doi: 10.1371/journal.pone.0309162 (PMC11426524; doi:10.1371/journal.pone.0309162)
Supplement: Attachment — Submitted filename: Response to Reviewers 20240624.docx [file pone.0309162.s004.docx]

June 24, 2024

Dr. Emily Chenette PhD, BA

Editor-in-Chief, *PLOS ONE*

Dear Dr. Chenette,

Thank you for the opportunity to revise and re-submit our article for consideration in *PLOS ONE*. Our article focuses on a systematic review protocol aimed at evaluating the effects of distance interventions intended to enhance preparedness among informal caregivers of older adults.

Each of the reviewer’s comments were most helpful, and the revisions have clarified and strengthened this paper. We have included each of the reviewer’s comments and the details of our responses below. Changes made in the manuscript are tracked.

All authors meet the authorship criteria outlined by the International Committee of Medical Journal Editors and agree to this submission. The manuscript is not and will not be submitted elsewhere for publication while under review at *PLOS ONE*. Neither the study co-authors nor I have competing interests or conflicts of interest in terms of any relationship with funders or other entities.

Please let me know if you have any questions. I look forward to hearing the review results.

Sincerely,

Fernanda L. F. Dal Pizzol, BScN, MN, PhD Candidate

University of Alberta, Faculty of Nursing

11405-87 Avenue

Level 3, Edmonton Clinic Health Academy

Edmonton, Alberta. Canada T6G 1C9

[fenglerd@ualberta.ca](about:blank)

We would like to express our gratitude to the reviewers for dedicating their time and providing helpful, constructive comments. Below, we provide detailed responses to each of the reviewers' comments. Changes made in the manuscript are tracked.

| **Reviewer 1** | | | |
| --- | --- | --- | --- |
| **Comment Number** | **Reviewer Comments** | **Our response** | **Pages** |
| 1 | In “Distance interventions for enhancing preparedness in informal caregivers of older adults: A systematic review protocol” and the authors explained the need for systematic review on this topic. I consider that the concepts described in this manuscript are worth studying but needs some revision.  The concept of “preparedness” is similar with “readiness.” However, I perceived the author's intentional use of this term. I would like a little more explanation on the intent of using this term and how it differs from readiness. | We have provided an explanation of how preparedness aligns with and differs from surrogate terms and related concepts in the background section of the protocol paper as suggested. | 5-6 |
| 2 | **Background:** In your background, I am not sure why you are focusing on the caregivers of the “older adult”. It looks like that your current background explains only the needs for the effect of distance interventions for informal caregiver. | You raised an important point, and we appreciate your bringing it to our attention. We have revised the background section to provide a clearer rationale for our focus on caregivers *of older adults*. | 5 |
| 3 | **Inclusion criteria:** You described inclusion criteria of your study as PICOS. However, I suggest you reconsider your study design framework (c, d). You defined your inclusion criteria of study design, dealing with only RCTs or quasi-experimental design. RCTs are designed with a randomly assigned comparison group, so that they always have a comparator. Then, it is not necessary to state whether or not there is a control group. Whereas, the quasi-experimental design implies multiple study designs, of which pre-post design is one. The section [d] describes two different concepts: whether there is a comparison group and the study design. Are interrupted time series analysis and quasi-RCTs eligible? I suggest that inclusion or exclusion be defined by a more detailed description of the study design. | We agree with your suggestions and have revised both our screening form and manuscript.  Both quasi-RCTs and interrupted time series designs are eligible for inclusion, as they are both quasi-experimental research designs that include a comparator (i.e., pre-post). | Please see our revised screening form: pages 1-3, especially letters e and f  Manuscript page: 9 |
| 4 | Inclusion criteria: You defined inclusion criteria of participant as “caregivers of older adults.” If the participants in the study you will review cross the age of 60 (e.g., 50-70), how would you cope with such a case? | As stated in our revised screening form, we will not include studies that aimed to involve older adults under the age of 60. | Please see our revised screening form: pages 2-3, letter c |
| 5 | Search protocol: You will use seven electronic databases however it does not contain trial registries. In a systematic review, I would suggest searching unpublished literature to minimize publication bias. | Our search yielded several trial registries and conference abstracts. However, we will only include studies that have provided a full report, whether as a peer-reviewed published study, pre-print, thesis, or dissertation. After reviewing the trial registries and conference abstracts resulting from our search, we couldn't confirm with 100% certainty whether they meet our inclusion criteria. Thus, we have decided to include only the complete reports to confirm if they meet the inclusion criteria, as the majority of our studies require thorough examination to ensure they meet all of our criteria. In addition, we aim to include only studies that reported quantitative findings as this is a requirement for our analysis. | Please see our revised screening form: page 3, letter e |
| 6 | Search strategies (Appendix3): You made search strategies as to your concern, but the concept of search strategies is too many so that your current search strategies in the row #1 might be missing out on important study. The Cochrane Handbook for systematic reviews indicates structure of a search strategy; “(i) terms to search for the health condition of interest, i.e. the population; (ii) terms to search for the intervention(s) evaluated; and (iii) terms to search for the types of study design to be included.” In the row #1 contains the way of measurement, using proximity operator. It provably rejects some noise but misses on important studies. If you want to reject some noise, I would recommend you add the row of study types in your search strategies, as Cochrane mentioned. | Our search strategy aligns closely with our inclusion and exclusion criteria, as outlined in the Cochrane Handbook. We included measurement terms because they are integral to defining our criteria for study relevance. Despite using these terms and the proximity operator, our search yielded 1,139 studies for screening, affirming that our approach has not restricted the breadth of our search, and we are confident that relevant studies have not been overlooked.  Narrowing our search solely to experimental and quasi-experimental study designs could potentially constrain our research, as these methodologies vary in description across different literature sources. Therefore, we prioritized the measurement aspect of 'preparedness' by employing adjacency operators rather than focusing exclusively on study design,  which is less specific. | N/A |
|  | **Reviewer 2** | | |
|  | **Reviewer Comments** | **Our response** | **Pages** |
| 7 | The research question is well-defined, and the inclusion and exclusion criteria are thorough and appropriate. | Thank you for your positive feedback. | N/A |
| 8 | Regarding the inclusion criterion "preparedness must be an intervention outcome," it raises the question that if the study did not specifically aim to intervene upon caregiver preparedness, but caregiver preparedness changed as a secondary outcome, will the study be excluded? | We will include studies either if they intend to assess preparedness as a primary or secondary outcome. We have added a clarification for that in our protocol. | 9 |
| 9 | For 4.2 search protocol, clarity is needed on the dates of the initial and any subsequent searches, ensuring that the latest search is up to date. | Our search was conducted in December 2023, and we included this information in section 4.2 as suggested. | 10 |
| 10 | Using “Citation Chaser” for reference and citation tracking of all included articles during data extraction is recommended. | Thank you for bringing the 'Citation Chaser' software to our attention. We now plan to utilize it for both backward and forward citation searching. | 10 |
| 11 | For data extraction, other information that might be meaningful to extract include year of publication, study purpose/objective, care-recipient diagnosis/severity, care-recipient age range (pediatric vs. non-pediatric), psychometrics of measurement tools/instruments that assess caregiver preparedness (if applicable), and analytical approach (if applicable). | We have clarified in the paper that we will extract this information. | 12-13 |
| 12 | You have briefly mentioned this in the paragraph right above your aim that “Future reviews should consider how specific components of distance interventions, such as educational materials and emotional support, impact outcomes. Additionally, they need to compare different distance approaches targeting informal caregivers of older people.” To address this gap, it could be helpful if you can briefly describe how the results will be organized in 4.10 Dissemination section. For example, is there a method that helps categorize different types of intervention (caregiver-focused intervention vs. dyadic intervention; education-related intervention vs., non-education related intervention; caregiver task-focused intervention vs., caregiver emotion-focused intervention)? It will be meaningful to compare and contrast the effectiveness of interventions within each category. | We have now incorporated this information into the 4.10 dissemination section as suggested. | 17-18 |
| 13 | While the study design, methodology, and writing style are satisfactory, there is a hesitance regarding the novelty of the topic. Session 2.1 “Why is this review needed in light of existing reviews” is not very persuasive. The three existing reviews (citation 16-18 in the manuscript) with similar topics are not very outdated (two in 2016, one in 2020). The three reviews focused on effectiveness of interventions for caregivers of patients with different diagnosis, including stroke, dementia, and heart failure. Although I agree with the author that including both formal and informal caregivers is a huge gap to address, we still need to question how helpful this review will add into the literature if we look at the effectiveness of interventions for caregivers for all kinds of diagnosis. The patient needs, caregiver routines, caregiver tasks, and emotional stress levels for different diagnosis can be very distinctive, which may lead to very different caregiver preparedness even with same intervention. Also, the previous studies “did not exclusively consider distance interventions approaches” does not seem like a strong limitation. To get the topic more focused and comparison more meaningful, we may suggest the author to consider focusing on only one type of caregivers (or caregivers with certain level of similarity besides age) but not sure if this is their goal. | The novelty of our review lies in our focus on evaluating effectiveness based on specific intervention components. Previous reviews offered limited discussion on how factors such as dosage and specific activities conducted during interventions influenced outcomes. Additionally, they did not compare different distance approaches for informal caregivers or assess their unique impacts on caregiver preparedness outcomes. These gaps have left the effectiveness of distance approaches uncertain. Our review aims to address these specific aspects and provide clarity on the effectiveness of distance interventions for informal caregivers. | 8 |
