# Supplementary material for: Distance interventions for enhancing preparedness in informal caregivers of older adults: A systematic review protocol
Source: PLoS One. 2024 Sep 26;19(9):e0309162. doi: 10.1371/journal.pone.0309162 (PMC11426524; doi:10.1371/journal.pone.0309162)
Supplement: Attachment — Submitted filename: Response to Reviewers 20240731.docx [file pone.0309162.s005.docx]

July 31, 2024

Dr. Emily Chenette PhD, BA

Editor-in-Chief, *PLOS ONE*

Dear Dr. Chenette,

Thank you for the opportunity to revise and re-submit our article for consideration in *PLOS ONE*. Our article focuses on a systematic review protocol aimed at evaluating the effects of distance interventions intended to enhance preparedness among informal caregivers of older adults.

Each of the reviewer’s comments were most helpful, and the revisions have clarified and strengthened this paper. We have included each of the reviewer’s comments and the details of our responses below. Changes made in the manuscript are tracked.

All authors meet the authorship criteria outlined by the International Committee of Medical Journal Editors and agree to this submission. The manuscript is not and will not be submitted elsewhere for publication while under review at *PLOS ONE*. Neither the study co-authors nor I have competing interests or conflicts of interest in terms of any relationship with funders or other entities.

Please let me know if you have any questions. I look forward to hearing the review results.

Sincerely,

Fernanda L. F. Dal Pizzol, BScN, MN, PhD Candidate

University of Alberta, Faculty of Nursing

11405-87 Avenue

Level 3, Edmonton Clinic Health Academy

Edmonton, Alberta. Canada T6G 1C9

[fenglerd@ualberta.ca](about:blank)

We would like to express our gratitude to the reviewers for dedicating their time and providing helpful, constructive comments. Below, we provide detailed responses to each of the reviewers' comments. Changes made in the manuscript are tracked.

| **Reviewer 1** | | | | |
| --- | --- | --- | --- | --- |
| **Comment Number** | **Reviewer Comments** | **Our response** | **Pages** |  |
| 1 | Thank you for your careful revision. However, I could not understand only one point I have already pointed out. I would like to ask you to reconsider this point.  I asked you as follows; #4 Inclusion criteria: You defined inclusion criteria of participant as “caregivers of older adults.” If the participants in the study you will review cross the age of 60 (e.g., 50-70), how would you cope with such a case? And then, your reply was as follows; As stated in our revised screening form, we will not include studies that aimed to involve older adults under the age of 60. I understood this inclusion criteria. However, if your review included a study that was not age-restricted and that study conducted a subgroup analysis by age, would the results of this subgroup analysis be covered in your study? Or how would they be treated? I am sorry, but I would appreciate additional clarification. | Thank you for your question and the opportunity to respond. Our intention is to include both experimental and quasi-experimental studies that have designed interventions specifically for informal caregivers of older adults.  With that being said, we have established criteria to ensure our focus remains on the intended demographic. Therefore, if a study includes care recipients who are 59 years of age or younger, it will be excluded from our review. This age criterion is essential to maintain the relevance and applicability of our findings to the population of older adults and their caregivers.  We have revised our wording to include this explanation in our screening form: “Only studies that include interventions designed for caregivers of *older adults* will be included. The age of 60 years was selected according to how the United Nations currently refers to ‘older’ people (United Nations, 2019). Therefore, care recipients must be at least 60 years of age. Studies that include care recipients who are 59 years of age or younger, even if they conduct a subgroup analysis by age, should be excluded”. | Please refer to letter C in our inclusion and exclusion form. |  |
|  | **Reviewer 2** | | |  |
|  | **Reviewer Comments** | **Our response** | **Pages** |  |
| 2 | Overall, I am pleased with the authors' response and believe this manuscript has the merit of being published. Here are a few suggestions for consideration. The authors do not need to address them now in the protocol, but the following could be helpful to think about for the full manuscript. | Thank you very much for your suggestions. Please see our responses to your suggestions and subsequent comments below. | N/A |  |
| 3 | Database Search: It might be beneficial to search in Sociology Source Ultimate (via EBSCOhost) because caregiver preparedness is a socially constructed concept. If this search does not yield additional articles, it can be ignored. Ultimately, the authors should decide if the current seven databases are sufficient to address the research question. | We don’t have access to that particular database. However, we agree that it is likely unnecessary to include an additional database, as CINAHL, PsycINFO, and Scopus already cover some journals in sociology. We are confident that the selected seven databases are sufficient to address our research question. | N/A |  |
| 4 | Search Update: The initial search occurred in December 2023. An update before the full manuscript publication would be helpful. According to the Cochrane Handbook, "The search must be rerun close to publication if the initial search date is more than 12 months (preferably 6 months) from the intended publication date, and the results screened for potentially eligible studies." | Thank you for raising this important point. We will run an updated search before the publication of our full report, and this information has been added to our protocol manuscript. | Manuscript p. 10 |  |
| 5 | Data Extraction: The columns for “information about caregiving” and “intervention” may contain large amounts of information, making synthesis difficult later. Consider breaking them down into more manageable pieces. These suggestions are intended to improve the final manuscript and are not required at this stage. | Thank you for your suggestion. We have separated our data extraction into two forms (i.e., study participants and study characteristics) to ensure that the information is more organized and easier to visualize. |  |  |
